# The Great Importance of Nourishment in Fever

**Published:** 1855-08

**Authors:** 


*The Great Importance of Nourishment in Fever.* By Dr. Stokes.

I wish, also, strongly to impress on you the great importance of the use of other forms of nourishment in this disease; for we must not only keep up the nervous energy of the system by wine, but we must support nature by food. There is no mistake more fatal in fever, than the withholding of food. I was early taught the importance of the use of careful nourishment in fever, by my friend and colleague, Dr. Graves. I remember once, Dr. Graves, when speaking of the necessity of the use of nourishment in fever, made use of these words: "If you are at a loss for an epitaph to be placed on my tomb, here is one for you—*He fed fevers.*" In addition to the prejudices with which the inflammatory doctrine imbued so many minds, with respect to the use of food in fever, there was a new set of arguments raised against it, in consequence of the experiments of an American physician. I allude to the case observed by Dr. Beaumont, and so often quoted since. In this remarkable case, various medicinal substances and articles of food were introduced through an external fistula into the stomach, their effects being noted, as also the conditions of temperature, vascularity, &c. A set of results were subsequently published in connection with the action of the stomach upon food. One of the results stated to have been thus obtained, was that the existence of the state of fever altogether suspended the process of digestion. Here was a statement which had the appearance of being the result of strict observation. It influenced a number of young men; but did it influence those who had once been in charge of a fever hospital? Not at all; because those men knew very well that, no matter what Beaumont might say about the stomach not digesting when the patient had fever, in thousands of cases patients in fever digested remarkably well, required food, and derived benefit from it. In a large number of cases of typhus fever, the stomach has an excellent power of digestion; and, I believe, if we were bold enough, we would find that many articles of food usually forbidden to fever patients, might be given to them with safety.—*Ibid.*

